# Relative clinical effectiveness of carbon ion radiotherapy: theoretical modelling for H&N tumours

**DOI:** 10.1093/jrr/rrv016

**Published:** 2015-04-08

**Authors:** Laura Antonovic, Alexandru Dasu, Yoshiya Furusawa, Iuliana Toma-Dasu

**Affiliations:** 1 Medical Radiation Physics, Department of Physics, Stockholm University, Stockholm, Sweden; 2 Department of Radiation Physics and Department of Medical and Health Sciences, Linköping University, Linköping, Sweden; 3 Next Generation Medical Physics Research Program and International Open Laboratories, National Institute of Radiological Sciences, Chiba 263–8555, Japan; 4 Medical Radiation Physics, Department of Oncology and Pathology, Karolinska Institutet, Stockholm, Sweden

**Keywords:** hypoxia, RBE, TCP, RCR, carbon ion, fractionation, RCE

## Abstract

Comparison of the efficiency of photon and carbon ion radiotherapy (RT) administered with the same number of fractions might be of limited clinical interest, since a wide range of fractionation patterns are used clinically today. Due to advanced photon treatment techniques, hypofractionation is becoming increasingly accepted for prostate and lung tumours, whereas patients with head and neck tumours still benefit from hyperfractionated treatments. In general, the number of fractions is considerably lower in carbon ion RT. A clinically relevant comparison would be between fractionation schedules that are optimal within each treatment modality category. In this *in silico* study, the relative clinical effectiveness (RCE) of carbon ions was investigated for human salivary gland tumours, assuming various radiation sensitivities related to their oxygenation. The results indicate that, for hypoxic tumours in the absence of reoxygenation, the RCE (defined as the ratio of *D*_50_ for photons to carbon ions) ranges from 3.5 to 5.7, corresponding to carbon ion treatments given in 36 and 3 fractions, respectively, and 30 fractions for photons. Assuming that interfraction local oxygenation changes take place, results for RCE are lower than that for an oxic tumour if only a few fractions of carbon ions are used. If the carbon ion treatment is given in more than 12 fractions, the RCE is larger for the hypoxic than for the well-oxygenated tumour. In conclusion, this study showed that *in silico* modelling enables the study of a wide range of factors in the clinical considerations and could be an important step towards individualisation of RT treatments.

## INTRODUCTION

Interest in using carbon ions for radiation therapy for malignant tumours has increased recently, based on the potential advantages with respect to dose conformation to the target, the increased relative biological effectiveness (RBE) and the decreased hypoxia-related resistance of this type of radiation in comparison with that of photons and other radiation with low linear energy transfer (LET). Consequently, an increasing number of clinical centres using carbon ion radiation therapy are in operation worldwide.

To a large extent, the potential advantages of carbon ion therapy have been identified in carefully designed laboratory experiments, in which influential factors were varied one at a time. For example, for a given cell line in a controlled microenvironment, the doses required to achieve a certain level of cell survival were determined for radiation of a given type with various LET [1]. The initial experimental observations were subsequently used to formulate pre-clinical hypotheses, which were further tested in carefully designed clinical trials. Similarly, the influence of the microenvironment was also determined for radiation with a given LET [2]. These types of experiments have allowed the quantification of many of the potential improvements that could be achieved with carbon ions in comparison with low-LET radiation.

Clinical situations, however, are much more complex than these experiments because they include a mixture of the factors that influence the final outcome. The interplay of clinically relevant factors should, therefore, be assessed as a global clinical effectiveness of the particle treatment in comparison with that of the best reference radiation treatment that is currently available for each cancer type under investigation. According to Tsujii *et al.* (2008) [3], the three most frequently treated conditions with carbon ion therapy are prostate, lung, and head and neck (H&N) cancers. For each of these three types of cancer, there is increasing evidence that the most efficient photon treatment would be delivered with non-standard fractionated schedules. The sensitivity to fractionation of prostate tumours has made them good candidates for extremely hypofractionated photon treatments [4]. Similarly, stereotactic body radiation therapy (SBRT) involving large doses per fraction has been successfully used in the treatment of lung tumours [5]. On the other hand, for rapidly growing H&N tumours, hyperfractionated treatments delivering less than the standard 2 Gy per fraction have great potential to increase local control [6]. For the carbon ion treatments, however, the tendency is to move towards giving the dose in only a few fractions of radiation. One would, therefore, have to determine the relative clinical effectiveness (RCE) that takes into account the complex clinical situation, including the various possible fractionation patterns [7]. A simple concept like the Relative Biological Effectiveness (RBE), defined as the ratio of the dose of reference low-LET radiation to the dose of the test high-LET radiation corresponding to the same biological effect, can only be used when the number of fractions is the same for the test and reference irradiation [8, 9]. Similarly, the isoeffective dose weighting factor, which has been proposed for the comparison of treatments involving different numbers of fractions [8], restricts by definition the reference arm to photons delivered in 2 Gy per fraction, and therefore is highly limited as well. A different concept would thus have to be used for the very likely case that hypofractionation is used for the carbon ion treatment and conventional fractionation is used for the photon treatment.

The present study aims to theoretically investigate the dependence of the more general RCE on various fractionation patterns for the test and the reference radiation, taking into account some of the key aspects of clinical relevance. These include the distributions of the heterogeneous dose and LET in the target volume, as well as the heterogeneous radiation-sensitivity of the cells in the tumour, related to their oxygenation.

## MATERIALS AND METHODS

**Fig. 1.**
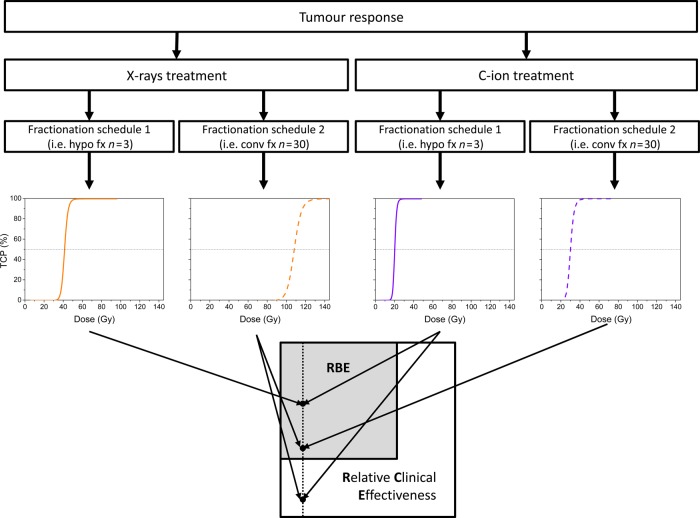
Schematic illustration of the study.

The design of the current study is graphically illustrated in Fig. 1. The response of *in silico* modelled tumours to X-ray or carbon ion treatments was determined in terms of tumour control probability (TCP). The number of fractions, *n*, was fixed for each dose–response curve, while the dose per fraction, *d*, was increased. In Fig. 1, one hypofractionated schedule (*n* = 3) and one conventionally fractionated schedule (*n* = 30) were assumed. X-rays were regarded as reference radiation and C-ions as test radiation.

The effectiveness of one of the treatment modalities relative to the other was assessed by calculating the ratio of doses corresponding to 50% TCP (*D*_50_) of the two modalities (*D*_50, ref_ for the reference radiation and *D*_50, test_ for the test radiation).

$$RCE=\frac{D_{50,\;\mathrm{ref}\;\;\;}}{D_{50,\;\mathrm{test}\;}}.$$

If the comparison was performed between the hypofractionated carbon-ion treatment and the conventionally fractionated X-ray treatment, the RCE was calculated as the ratio of *D*_50_ values in the dose–response curves in panels 3 and 2 in Fig. 1, as indicated by the arrows.

If the comparison was made between the hypofractionated carbon-ion treatment and the hypofractionated X-ray treatment, the RCE value is indicated by the corresponding two arrows. In this case, the numbers of fractions are the same for the test and the reference radiation, and the RCE would be the same as the RBE. The same holds true for the comparison between two conventionally fractionated schedules. The RBE is a particular case of the RCE and can be written as follows, considering also the fact that the test and reference irradiation conditions are non-identical with respect to dose and LET distribution:

$$RBE=(RCE{)}_{\mathrm{iso}-n}.$$

For simplicity, *n*_test_ and *n*_ref_, for carbon ions and X-rays, respectively, can be used to indicate the numbers of fractions used for assessing the *D*_50_ for each radiation type. The RCE for the numbers of fractions, *n*_test_ and *n*_ref_, would thus be written as $RCE_{n_{\mathrm{test}}}^{n_{\mathrm{ref}}}$.

The details of the individual steps in the calculation of the RCE are given in the following sections.

### Simulation of the *in silico* tumours

A previously developed model was used to simulate virtual tumours with respect to size, shape, density of clonogenic cells, and oxygenation. The model has been extensively described in previous publications by the authors [10–13]. The model is based on a Monte Carlo method used to generate well or poorly oxygenated tumour regions according to variable densities of the capillaries, with mean intervessel distances of between 100 and 160 μm. The capillaries were assumed to originate on the venous side of the vasculature and, therefore, to have low oxygen content, distributed around 40 mmHg. The oxygenation of the tumour cells was calculated numerically, assuming a diffusion coefficient of 2 × 10^−5^ cm^2^ s^−1^ in the tissue and a maximum oxygen consumption rate of 15 mmHg s^−1^. These are generic parameters previously shown to describe the oxygenation of a wide range of tumours [14].

**Fig. 2.**
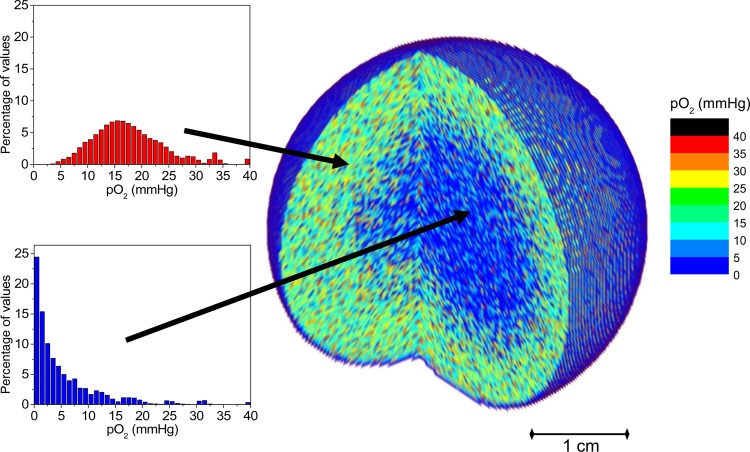
Illustration of the *in silico* tumour used in the study.

For the present study, spherical tumours (4.0 cm in diameter) were considered. The total number of clonogenic cells in the tumour was assumed to be 10^6^. The clonogenic cells were assumed to be homogeneously distributed throughout the tumour volume. The distribution of oxygen partial pressure within the tumour was based on the above-mentioned oxygenation model, in which the oxygen diffusion from the blood vessels and the oxygen consumption by the cells were taken into account [10–13]. Depending on the density of blood vessels within the tumour, the resulting oxygen distribution would be representative of either well-oxygenated or hypoxic tumours. An illustration of the size, the shape and the oxygen distribution of a virtual tumour used in this study is shown in Fig. 2.

Two representative tumour cases were considered in this study. One was a tumour presenting a hypoxic core and a rim of well-oxygenated cells around it, as illustrated in Fig. 2. The distributions of oxygen partial pressure in the two regions are shown as histograms. The second case was a well-oxygenated tumour similar to the one in Fig. 2, but without the hypoxic core. The oxygenation in the entire tumour is, therefore, described by the red histograms in Fig. 2 (upper panel).

Changes in the oxygenation of the hypoxic tumour related to the fluctuations in acute hypoxia that might occur between fractions were also included in the simulations. The simulation details of such local oxygenation changes (LOCs) are given elsewhere [13, 15].

### Simulation of the C-ion and X-ray treatments

A carbon ion treatment plan was made, in which the 4-cm *in silico* tumour constituted the clinical target volume (CTV), and a planning target volume (PTV) was created by isotropic expansion of the CTV by 0.5 cm. The carbon ion treatment was given using only one beam. The plan was optimized using TRiP (TReatment planning for Particles), the treatment planning system used at the GSI (Gesellschaft für Schwerionenforschung), using the generic input parameters in the Local Effect Model I (LEMI) implemented in TRiP (α = 0.1 Gy^−1^, β = 0.05 Gy^−2^ and D_t_ = 30 Gy) [16]. The resulting physical dose and dose-averaged LET-distributions were heterogeneous over the tumour volume, with doses ranging from 80 to 120% of the prescribed dose to the centre of the PTV and LETs varying between 30 keV/µm (at the proximal edge of the CTV relative to the beam entrance) and 90 keV/µm (at the distal edge of the CTV).

The corresponding photon plan was simulated as delivering a homogenous dose over the target, i.e., all the voxels would receive the prescribed dose, corresponding to a homogeneity index of 0, as defined in ICRU Report 83 [17].

### Calculation of tumour control probability

The outcome of the treatment of the *in silico* tumours with carbon ions or with photons was assessed as TCP dose–response curves for fractionated schedules with varying numbers of fractions.

The dose–response curves were plotted as the calculated TCP for photon or carbon ion treatment, against the prescribed photon dose or the physical dose at the centre of the PTV for the carbon ion plan, respectively.

The TCP was calculated using a Poisson model accounting for the fractionation of the treatment, the distribution in dose, and also LET for the carbon ion treatment, as well as for the distribution of radiation sensitivity, as described by Equation 1:

(1) $$TCP=\exp\left\{-\sum_{i=1}^{N_{\mathrm{vox}}}N_{i}\prod_{\;j=1}^{n}S_{i,j}\left(d,L,\;pO_{2}\right)\right\},$$

where *N*_vox_ is the total number of voxels in the *in silico* tumour, *N_i_* is the number of cells in voxel *i* and $S_{i,j}(d,L,\;p{\text{O}}_{2})$ is the surviving fraction in voxel *i* at fraction *j* with dose *d*, the oxygen partial pressure *p*O_2_, and for C-ions, the LET *L*. The total number of clonogenic cells in the tumour was set to 10^6^ in all simulations.

The model chosen for the calculation of the surviving fraction of cells per voxel is the LET-parameterized repairable–conditionally repairable damage (RCR) cell-survival model adapted to account for oxygenation [15, 18]. The general expression for cell survival according to the current version of the advanced parameterized RCR model is given by Equation 2:

(2) $$S(d,L,\;p{\text{O}}_{2})=e^{-a(L)d/\tilde{O}(L,\;pO_{2})}+b(L)d/\tilde{O}(L,\;p{\text{O}}_{2})e^{-c(L)d/\tilde{O}(L,\;pO_{2})}$$

The parameters of the model relevant for carbon ions and their corresponding LET dependence were determined in a previous study based on cell survival data from published experiments on human salivary gland (HSG) tumour cells irradiated with carbon ions in the LET range of 22.5–501.5 keV/μm [2, 15].

The parameters of the model that would describe survival after low-LET photon-irradiation were also determined by fitting the corresponding data from the same dataset published by Furusawa *et al.* in 2000 [2].

The choice of the HSG tumour cell line for determining the radiobiological parameters required by the cell survival model was motivated by the fact that the data of this cell line is currently used clinically in the treatment planning system for optimizing the biologically equivalent dose distributions at the heavy-ion medical accelerator complex (HIMAC) at the National Institute of Radiological Sciences (NIRS) in Chiba and at the Gunma University, both in Japan [19].

One hypofractionated and one conventionally fractionated schedule were used, as previously mentioned. For carbon ion radiotherapy, the numbers of fractions were also varied in the range from extreme hypofractionation (using only three fractions) to a schedule involving 36 fractions of radiation (i.e. *n*_test_ = 3, 6, 9, 12, 15, 18, 21, 24, 30 and 36 and *n*_ref_ = 3 and 30).

## RESULTS AND DISCUSSIONS

**Fig. 3.**
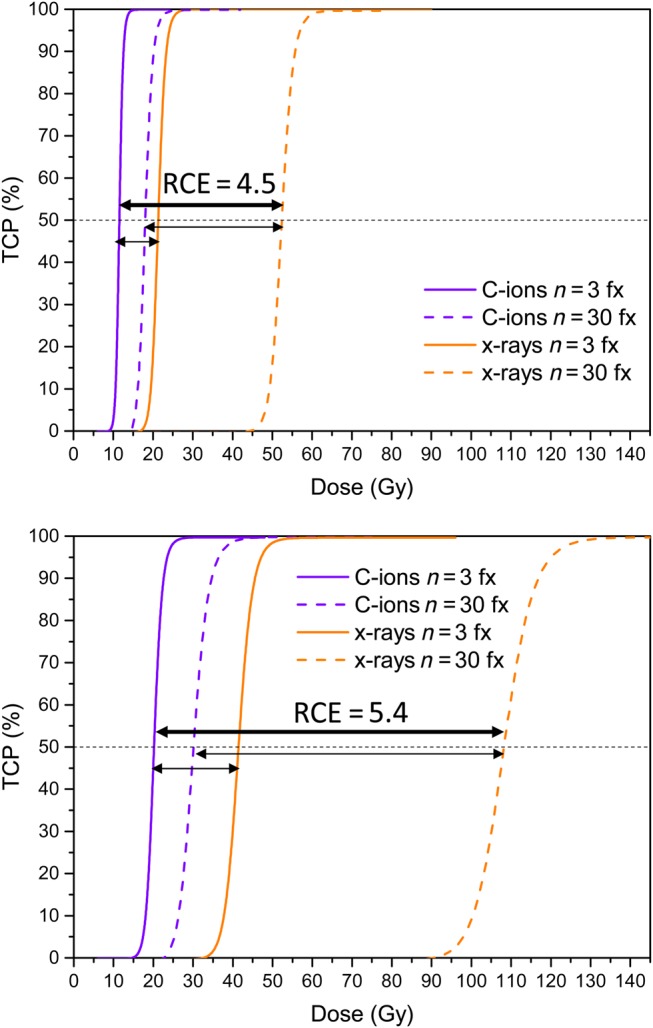
Dose–response curves for the well-oxygenated tumour (upper panel) and for the hypoxic tumour (lower panel). The different curves correspond to the irradiation with photons (orange curves) and to the irradiation with carbon ions (purple curves), with the total dose delivered in 3 fractions (solid lines) or in 30 fractions (dashed lines). The thin arrows pointing to the curves corresponding to the same number of fractions illustrate the RBE calculated as the ratio of *D*_50_ values. The RCE (calculated as the ratio of *D*_50_ values for the carbon treatment given in 3 fractions and the *D*_50_ values for the photon treatment given in 30 fractions) was 4.5 for the well-oxygenated tumour and 5.4 for the hypoxic tumour.

The results of the study for several representative cases are shown in Fig. 3. The dose–response after irradiation is shown in the upper panel of Fig. 3 for the well-oxygenated *in silico* tumour. The set of four curves corresponds to the two radiation modalities (photons and C ions) and the two fractionation-schedules (*n* = 3 and *n* = 30) used for each of them. The corresponding dose–response curves for the hypoxic tumour (assuming no reoxygenation) are shown in the lower panel of Fig. 3. As expected, the well-oxygenated tumour is more sensitive to radiation than the hypoxic one. This is reflected in the lower doses required for a certain level of tumour control.

The values of *D*_50_ for the various cases are presented in Table 1. These values were used to compute the RCE, as indicated by the arrows in Fig. 3.

**Table 1.** *D*_50_ values for *n* = 3 and *n* = 30 for oxic and hypoxic tumours (with LOCs and with static oxygenation)

	Well-oxygenated tumour	Hypoxic tumour	
		With LOCs	Static
***D*_50, C_**	11.5 Gy /3 fx	15.6 Gy /3 fx	20.2 Gy /3 fx
***D*_50, X_**	21.3 Gy /3 fx	31.2 Gy /3 fx	41.4 Gy /3 fx
***D*_50, C_**	18.0 Gy /30 fx	20.4 Gy /30 fx	30.1 Gy /30 fx
***D*_50, X_**	52.4 Gy /30 fx	61.6 Gy /30 fx	108.2 Gy /30 fx

The relatively high $RCE_{3}^{30}$ of 4.5 and 5.4 for the well-oxygenated and hypoxic tumour, respectively, includes both the effect of using different radiation qualities and treatment modalities as well as using different fractionation schedules for the different modalities. The separation of the influence of the two factors is shown in Table 2. The influence of radiation quality is described by the RCE, using the same number of fractions for the C ions and for the photon treatments ($RCE_{3}^{3}$ and $RCE_{30}^{30}$). On the other hand, the influence of fractionation is described by a fractionation ratio, $R_{3}^{30}$, which is the ratio of *D*_50_ for a treatment using 30 fractions to that of a treatment using 3 fractions when both treatments are of the same modality. The $RCE_{3}^{30}$ could thus be expressed as the product of the $RCE_{30}^{30}$ and the fractionation ratio $R_{3}^{30}$for C ions:

$$RCE_{3}^{30}=(R_{3}^{30}{)}_{C}\;RCE_{30}^{30}.$$

**Table 2.** The separate contributions to the RCE

	Well-oxygenated tumour	Hypoxic tumour	
		With LOCs	Static
${RCE}_{3}^{3}$	1.8	2.0	2.0
${RCE}_{30}^{30}$	2.9	3.0	3.6
$R_{3}^{30}$ **X-rays**	2.5	2.0	2.6
$R_{3}^{30}$ **C ions**	1.6	1.3	1.5
***OER* for C-ions with *n* = *3***	1	1.4	1.7
***OER* for C-ions with *n* = *30***	1	1.1	1.7
***OER* for X-rays with *n* = *3***	1	1.5	1.9
***OER* for X-rays with *n* = *30***	1	1.2	2.1

The effect of treatment modality is described by the RCE with equal numbers of fractions for test and reference radiation. The effect of fractionation is described by the fractionation ratio and the effect of tumour oxygenation by the OER.

In addition to the separate influence of radiation quality and fractionation, the influence of the tumour microenvironment was added to Table 2, expressed as the oxygen enhancement ratio (OER) for the various fractionation schedules and radiation qualities. According to the values in Table 2, the effect of using C ions instead of photons is the strongest effect, especially for many fractions, since fractionation has a larger impact on low-LET radiation compared with its impact on high-LET radiation. The $RCE_{30}^{30}$ ranges from 2.9 to 3.6, depending on tumour oxygenation. The larger fractionation sensitivity of photon treatments is also reflected in the higher fractionation ratios for photon treatments compared with C ion treatments. The $R_{3}^{30}$ for photons ranges from 2.0 to 2.5.

**Fig. 4.**
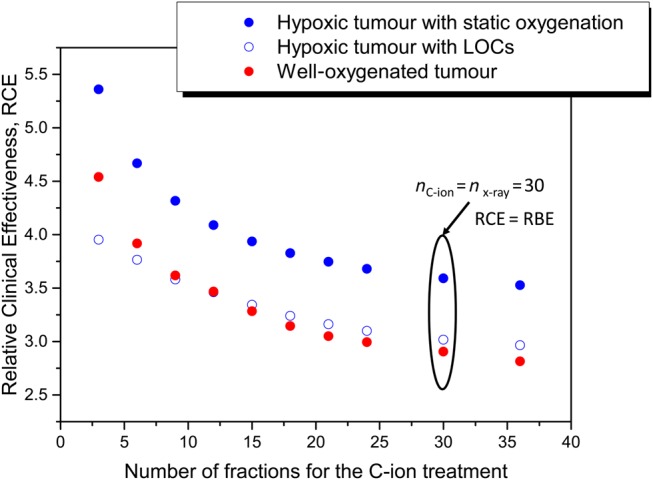
The relative clinical effectiveness of the carbon ion treatment delivered in the number of fractions indicated on the horizontal axis. The number of fractions for the photon treatment that is the reference radiation is 30.

Figure 4 shows the RCE for the range of fractionation schedules used for the carbon ion treatments (*n*_test_ = 3–36) when the reference photon treatment was delivered in 30 fractions. The RCE is shown for the well-oxygenated tumour as well as for the hypoxic tumour with and without reoxygenation. The results show an increased RCE when the number of C-ion fractions is decreased, which is expected due to decreased possibilities for repair of sublethal damage between fractions. The largest RCE is found for hypoxic tumours with static oxygenation, supporting the predictions and clinical findings that carbon ion therapy would have a great impact on tumours with poor oxygenation [20].

Interestingly, however, for hypoxic tumours with a large potential for LOCs, the RCE is considerably lower and comparable with the RCE for well-oxygenated tumours. For hypofractionated C-ion schedules, the RCE is even lower for the hypoxic tumour compared with that of the well-oxygenated tumour if LOCs are assumed. This is due to the decreased number of possibilities for LOCs in the C-ion treatment, leading to an increase in the dose required to control the tumours, while the number of possibilities is higher in the standard photon schedule. This illustrates the fine balance between radiosensitivity and reoxygenation, even for carbon ion therapy.

It should be mentioned that the findings in Fig. 4 have to be analysed in conjunction with the clinically relevant oxygen enhancement ratio corresponding to each fractionation schedule (discussed in a previous publication [15]). Although hypofractionated schedules show the highest relative effectiveness against persistently hypoxic tumours, hypoxic tumours still require a considerably higher dose for a certain TCP level. This is best illustrated by the OER values in Table 2, which are well above one for both statically hypoxic tumours and tumours with LOCs.

The results in Fig. 4 also show that there is no optimal fractionation schedule. Thus, hypofractionated treatments appear to be most effective against tumours with persistent hypoxia, but the advantage appears to be diminished for tumours with dynamic hypoxia, for which increasing the number of LOC opportunities leads to a reduction in the dose needed to control them. Furthermore, the best clinical gain would be obtained from the optimization of the treatment with respect to both tumour and normal tissue responses.

The results of this study show the power of *in silico* simulations for predicting responses to fractionated carbon ion therapy. These sorts of simulations could easily be used to understand the range of responses that could be expected from tumours with various oxygenations, as well as for the strategies needed to achieve optimal clinical responses. It has to be mentioned, however, that the reported values for the RCE would obviously depend on the actual simulation conditions, and therefore the present study would like to focus the discussion rather on the observed trends than the actual values for the RCE. The reported values are based on the physical dose of carbon ions to the centre of the PTV and on the dose-averaged LET, with a given spectrum corresponding to a given irradiation technique. Since the physical dose as well as the LET spectrum will differ for tumours of different sizes and irradiation geometries, the RCE values will also be different, but the trend of the variation is expected to be the same as is reported here. The RCE values will also change when different scenarios for the changes in the tumour oxygenation are considered. The effect of the treatment on the tumour oxygenation, resulting in changes to both acute and chronic hypoxia, might lead to a gradual improvement in the overall oxygenation of the tumour, depending on the size of the dose per fraction and the duration of the treatment. This would add an extra layer of complexity to the assessment of the RCE.

## CONCLUSION

The results of this study show that *in silico* modelling could be an important step for true individualisation of radiation treatment, because it allows the exploration of the wide variety of therapeutic options available, depending on the individual features of the patients. The findings of the present study should be further analysed and compared with the clinical outcome of treatments performed with different fractionation schedules on tumours with known oxygenation as a further step towards customised radiation therapy.

## FUNDING

Financial support from Radiumhemmets Forskningsfonder is gratefully acknowledged. Funding to pay the Open Access publication charges for this article was provided by Radiumhemmets Forskningsfonder, Stockholm, Sweden.
